# A role for midline and intralaminar thalamus in the associative blocking of Pavlovian fear conditioning

**DOI:** 10.3389/fnbeh.2014.00148

**Published:** 2014-05-01

**Authors:** Auntora Sengupta, Gavan P. McNally

**Affiliations:** School of Psychology, University of New South WalesSydney, NSW, Australia

**Keywords:** prediction error, Pavlovian fear conditioning, learning, aversive, intralaminar thalamus

## Abstract

Fear learning occurs in response to positive prediction error, when the expected outcome of a conditioning trial exceeds that predicted by the conditioned stimuli present. This role for error in Pavlovian association formation is best exemplified by the phenomenon of associative blocking, whereby prior fear conditioning of conditioned stimulus (CS) A is able to prevent learning to CSB when they are conditioned in compound. The midline and intralaminar thalamic nuclei (MIT) are well-placed to contribute to fear prediction error because they receive extensive projections from the midbrain periaqueductal gray—which has a key role in fear prediction error—and project extensively to prefrontal cortex and amygdala. Here we used an associative blocking design to study the role of MIT in fear learning. In Stage I rats were trained to fear CSA via pairings with shock. In Stage II rats received compound fear conditioning of CSAB paired with shock. On test, rats that received Stage I training expressed less fear to CSB relative to control rats that did not receive this training. Microinjection of bupivacaine into MIT prior to Stage II training had no effect on the expression of fear during Stage II and had no effect on fear learning in controls, but prevented associative blocking and so enabled fear learning to CSB. These results show an important role for MIT in predictive fear learning and are discussed with reference to previous findings implicating the midline and posterior intralaminar thalamus in fear learning and fear responding.

## Introduction

Pavlovian fear conditioning involves the learning of predictive relationships between a conditioned stimulus (CS) and an aversive unconditioned stimulus (US). As a consequence of this learning, subsequent presentations of the CS alone elicit a constellation of co-ordinated fear responses, including species-specific defense responses, potentiated startle, autonomic and endocrine responses. Both fear learning and fear responding depend on the amygdala, and the relevant intra-amygdala mechanisms are increasingly well-understood. In general, the basolateral amygdala (BLA) is critical for fear learning whereas the central amygdala (CeA) is critical for both fear learning and fear responding (Killcross et al., 1997; Maren and Quirk, 2004). Principal cells of lateral and basolateral nuclei (BLA) receive glutamatergic inputs from thalamus and cortex conveying information about the CS and US (Sah et al., 2008, 2003; Marek et al., 2013). These neurons form fear memories in an NMDA receptor-dependent manner (Schafe et al., 2001; Maren and Quirk, 2004). A network of GABAergic interneurons regulates activity of these cells and fear learning (Marek et al., 2013). BLA principal neurons project to CeA where inhibitory microcircuits control fear expression. The CeA has medial (CeAm) and lateral (CeAl) regions. CeAl neurons receive inputs from BLA principal neurons as well as other CeAl neurons. CeAl output neurons tonically inhibit brainstem projecting-CeAm neurons and removal of this inhibition activates CeAm neurons causing fear expression (Ciocchi et al., 2010; Haubensak et al., 2010; Li et al., 2013).

The actions of prediction error are central to this fear learning (Rescorla and Wagner, 1972). Animals learn to fear a CS in response to positive prediction error that is when the actual US on a conditioning trial exceeds the expected US. Likewise, animals will reduce fear to a CS in response to negative prediction error that is when the actual US on a conditioning trial is less than that expected. The critical role of prediction error in governing Pavlovian association formation is demonstrated through associative blocking (Kamin, 1968). In a blocking experiment, an experimental group is trained to fear CSA in Stage I via pairings with an aversive US, such as footshock. In Stage II, the blocking group receives compound training of CSA and CSB (AB) paired with footshock. Compared to a group that does not receive Stage I training, fear learning to CSB is prevented. The absence of positive prediction error in Stage II blocks fear learning to CSB. Although amygdala mechanisms for synaptic plasticity are sensitive to the actions of prediction error (Bauer et al., 2001; Johansen et al., 2010), how this sensitivity is achieved remains poorly understood. There is compelling evidence that vlPAG is involved in this process. For example, vlPAG microinjections of a μ-opioid receptor antagonist (MOR) augment fear learning in response to positive prediction errors (Cole and McNally, 2008) but impair fear learning in response to negative prediction errors (McNally et al., 2004; Cole and McNally, 2008). This manipulation also prevents the associative blocking of fear learning. For example, vlPAG microinjection of the MOR antagonist CTAP prior to Stage II of a blocking procedure prevents associative blocking, thereby enabling fear acquisition to the blocked CS (McNally and Cole, 2006). The midline and intralaminar thalamus (MIT)—including the paraventricular thalamus (PVT), rhomboid nucleus (Rh), reuniens nucleus (Re), and centromedial nucleus (CM)—receive dense ascending projections from vlPAG neurons, and, in turn, project extensively to prefrontal cortex (Krout et al., 1998). Evidence from both rodent and human imaging studies suggests that MIT may be important in fear prediction errors. For example, in humans, the magnitude of the thalamic fMRI BOLD elicited by an aversive US is related to the degree to which that US is expected (Dunsmoor et al., 2008). In rats, expression of the activity marker c-Fos in several MIT regions, including CM, Rh, and Re during fear conditioning correlates with the magnitude of positive prediction error and hence the amount of fear learning (Furlong et al., 2010). Moreover, this same relationship between activity marker expression and positive prediction error is observed in CM neurons retrogradely labeled from dmPFC, strongly suggesting that an ascending pathway from MIT to dmPFC is important for regulating fear learning. Taken together, these findings have led to our suggestion that fear prediction errors are conveyed to amygdala and cortical learning networks by an ascending circuitry involving the vlPAG and MIT (McNally et al., 2011). However, the existing evidence supporting a role for the MIT in predictive fear learning is correlational and a causal role is yet to be demonstrated.

The aim of this experiment was to investigate the role of the MIT in Pavlovian fear conditioning, specifically in predictive fear learning, using conditioned suppression as the measure of learned fear. To do so, we used an associative blocking design and reversible inactivation of the MIT via infusions of the sodium channel blocker bupivacaine hydrochloride. Local administration of bupivacaine hydrochloride can produce a dose dependent blockade of synaptic transmission via depression in postsynaptic cell activity and axonal conduction as well as decrements in neurotransmitter release. For the purposes of this procedure bupivacaine hydrochloride was specifically chosen not only for its mechanism of action but also its duration of action (Tabatabai and Booth, 1990) which should be sufficient for the 70 min training sessions used in the blocking design. The design was a 2 × 2 factorial. The first factor was the type of Stage I fear conditioning training (Control or Block). Groups Block received Stage I fear conditioning involving pairings of CSA with a shock US whereas groups Control did not. In Stage II, groups Block and Control both received fear conditioning involving pairings of a compound CSAB with a shock US. The second factor was type of infusion into the MIT prior to Stage II training (Bupivacaine or Saline). Both groups were then tested for their fear responses to CSB. Prior fear conditioning of CSA should block fear learning to CSB for group Block-Saline. The questions of interest here were: (1) is the MIT necessary for fear learning, and hence what effect does reversible inactivation of the MIT have on fear learning to CSB in group Control-Bupivacaine? And, (2) is the MIT necessary for predictive fear learning, and hence what effect does reversible inactivation of the MIT have on the blocking of fear learning to CSB in group Block-Bupivacaine?

## Materials and methods

### Subjects

Subjects were 34 experimentally naive male Wistar rats obtained from the Animal Resource Centre (Perth, Australia). All rats were housed in groups of 8 maximum in a colony room maintained on a 12 h light-dark cycle (lights on at 7 am). Prior to the experiment, rats had free access to water. During the experiment rats had free access to water and were maintained at 90% of their free feeding weight. Rats were handled prior to the commencement of the experiment to reduce handling stress. Procedures were approved by the Animal Care and Ethics Committee (University of New South Wales) and conducted with respect to National Institutes of Health (NIH) guidelines in *Guide for the Care and Use of Laboratory Animals* (NIH Publications No. 80-23, 1996).

### Surgery

Rats received intraperitoneal injections (i.p.) with a mixture of 1.3 ml/kg ketamine anesthetic (Ketapex; Apex Laboratories, Sydney, Australia) at a concentration of 1.0 mg/ml and 0.3 ml/kg of the muscle relaxant xylazine (Rompun; Bayer, Sydney, Australia) at a concentration of 20 mg/ml. After being shaved to expose the skin surface, each rat was placed in the stereotaxic apparatus (Model 900, Kopf, Tujunga, CA), with the incisor bar maintained at −3.3 m. A hand drill was used to expose the brain surface and a 6 mm 26 gauge guide cannula was inserted into the MIT, targeted at the CM, A-P: −2.6; M-L: 0.0; D-V: −5.6; all co-ordinates in mm from Bregma according to the atlas of Paxinos and Watson (2007). The cannula was held in place with dental cement and four jewelers screws attached to the skull. A dummy cannula remained inserted in the guide cannula for the duration of the behavioral procedure, excluding infusion days, to prevent occlusions in the guide cannula. Following surgery, rats received an intramuscular injection of 0.15 ml of a 3.0 mg/ml solution of procaine penicillin, 0.1 ml of 1.0 mg/ml cephazolin sodium, and s.c. injection of 5 mg/kg carprofen. Rats were allowed 5 days to recover from surgery prior to commencement of training, during which time they were monitored daily.

### Apparatus

Training and testing took place in 8 identical Med Associates (St. Albans, VT, USA) chambers with the dimensions: 24 (length) × 30 (width) × 21 (height) cm. The chambers were constructed of Perspex top and rear walls as well as a Perspex hinged door. The sidewalls were constructed of stainless steel. The grid floor was built of steel rods, 4 mm in diameter, and spaced 15 mm apart. The grid floor was connected to a constant-current generator. The left sidewall was equipped with a magazine (5 × 5 cm entry space with a dish), which was connected to a pellet delivery system. A retractable lever, 4 cm to the left of the magazine hopper delivered a 45 mg grain pellet (Able Scientific Biotechnology, Western Australia). All chambers were illuminated for the testing duration with a house-light mounted on the top right side wall. Each chamber was placed in a slightly larger soundproof box with dimensions 83 (length) × 59 (width) × 59 (height) cm. A fan was attached to the interior of the box to provide sufficient ventilation.

A 60 s 80-dB white noise acted as CSA. This CS was delivered through a speaker attached to the right side wall of the chamber. Two key lights, both 24 mm in diameter, mounted either side of the magazine hopper, 14 cm apart, delivered a flashing light of 60 s duration that served as CSB. The US was a 1 s 0.8 mA scrambled footshock delivered through the grid floor. The delivery of all events was controlled by Med PC IV computer software (Med Associates, St Albans, VT, USA). After behavioral testing, rats were returned to their home cages and the testing chambers were cleaned with 80% ethanol solution. Corn-cob bedding underneath the grid floor was changed after every testing session.

### Procedure

#### Magazine training

On Days 1 and 2, rats received magazine training whereby pellets were delivered every 120 s, regardless of the rat's behavior. Each lever press was also reinforced with pellet delivery. The session ended after 1 h or after rats reached 100 lever presses. Animals that did not respond 100 times for pellets by the end of the second session were hand shaped.

#### Lever press training

Over the next 8 days rats received lever press training on variable interval (VI) schedules. On Day 3 rats were trained on a VI30 schedule for 1 h. From Day 4 to the end of the experiment, rats were maintained on a VI120 schedule which, unless otherwise noted, was 2 h in duration. On Day 11 rats received non-reinforced presentations of CSA and CSB superimposed over the VI120 schedule. Each CS was presented 4 times, in a random order at an average inter-stimulus interval of 15 min. This was done to familiarize the rats with the CSs.

#### Stage I

On Day 12, rats were randomly allocated to receive Stage I training (group Block) or further lever press training on a VI120 schedule (group Control). Stage I training involved three days of conditioning. Group Block received four presentations of CSA co-terminating with a 1 s 0.8 mA footshock US. The intertrial interval (ITI) was on average 30 min. Each session lasted for 2 h.

#### Stage II

On Days 15 and 16, all rats received Stage II training. Immediately prior to each day of Stage II training, rats received 0.5 μL infusions of either 0.5% (wt/vol) Bupivacaine hydrochloride or 0.9% (wt/vol) saline directed at the MIT. Dummy caps were removed and a 33-gauge microinjection cannula was inserted into the guide cannula, targeting 1 mm below the tip of the guide cannula. The microinjection cannula was attached to a 10 μL Hamilton glass syringe by PE-50 tubing. The glass syringe was operated by an infusion pump (KD Scientific) that infused each solution at 0.25 μL/min. The microinjection cannula was left in place for a further 2 min to permit diffusion into neural tissue. Rats were then placed in the conditioning boxes for Stage II training. During this training, rats received four presentations of CSAB compound co-terminating with the US. The ITI was on average 15 min. Stage II training sessions were 70 min in duration.

#### Test

On Day 17 rats were tested. During this 70 min test, CSB was presented 4 times. ITI was on average 15 min.

### Histology

At the conclusion of testing, rats were given an overdose of sodium pentobarbital and their brains were removed and frozen at −18°. Unfixed brains were sectioned coronally at 40 μm through the MIT. Every section through the MIT was collected on glass slides, and subsequently stained with Cresyl violet. Slides were coverslipped using the mounting agent Entellan. Cannula placements were verified at the microscope using the boundaries defined by Paxinos and Watson (2007).

### Data analysis

Lever pressing during each day of the experiment was recorded. In these and remaining experiments, suppression ratios (SR) were calculated as *SR* = *c*/(*c* + *d*) (Annau and Kamin, 1961). Where *c* = lever presses during CS and *d* = lever presses made in the minute prior to the CS (the preCS period). A suppression ratio of 0.5 indicates no fear (no differences in preCS vs. CS lever pressing) and a suppression ratio of 0 indicates asymptotic fear. Suppression ratios for the first CS presentation each day were analyzed using orthogonal contrasts and the Type I error rate was controlled at 0.05 level using the planned orthogonal contrasts.

## Results

### Histology

**Figure 1:**
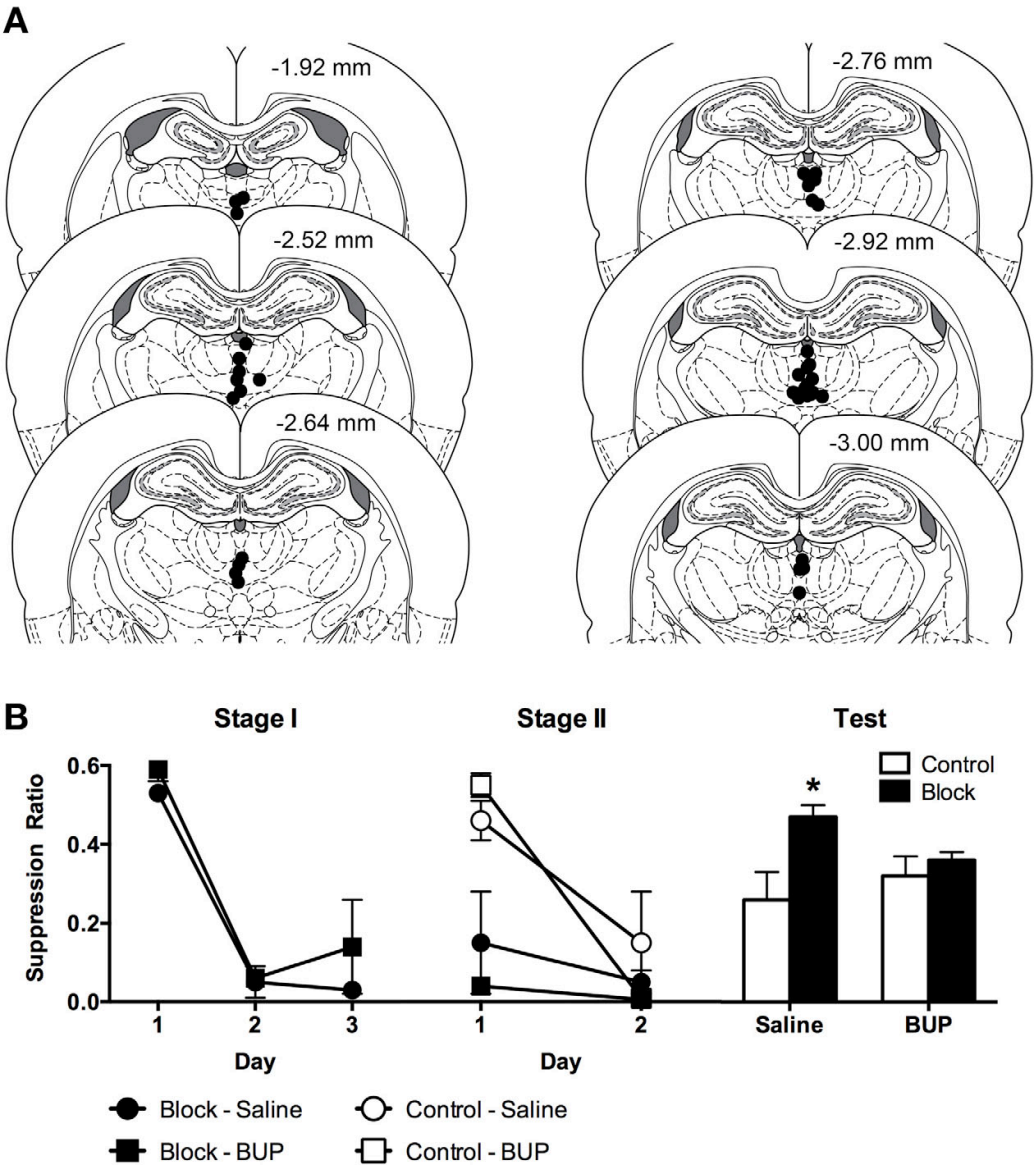
**(A)** Cannula placements in the midline thalamus according to the atlas of Paxinos and Watson (2007). Each circle represents the ventral tip of the microinjection cannulae. **(B)** Mean and SEM suppression ratios across Stage I, Stage II, and Test. *Indicates significant difference (*p* < 0.05) compared to Control-Saline.

Cannula placements, depicting the ventral most tip of the microinjection cannulae are provided in Figure 1A. Data from 5 rats were excluded because placements were lateral from the midline region, bordering on the mediodorsal thalamus and the habenular complex. All other cannulae were located in the MIT. The final numbers per group, after histological verification of cannula location, were: Block-Bupivacaine: *n* = 8; Block-Saline: *n* = 7; Control-Bupivacaine: *n* = 7 and Control-Saline: *n* = 7.

### Precs lever pressing

Groups Block-Bupivacaine (*M* = 20.07, *SD* = 7.40), Block-Saline (*M* = 18.52, *SD* = 7.29), Control-Bupivacaine (*M* = 2 0.85, *SD* = 7.81), and Control-Saline (*M* = 22.10, *SD* = 12.58) did not differ on average lever pressing across the session on the last day of VI120 training, *Fs*_(1, 25)_ < 1, *p* > 0.05. Lever presses following pre-exposure were not significantly different between groups, *Fs*_(1, 14)_ < 2, *p* >0.05.

The mean and standard error of the mean (SEM) pre-CS lever presses across all days of Stage I, Stage II, and test are shown in Table 1. There were no differences in Stage I pre-CS lever pressing between Block-Bupivacaine and Block-Saline averaging across days of training, *F*_(1, 13)_ = 1.44, *p* > 0.05. Furthermore, there was no significant difference in pre-CS lever presses across training days when averaged across Group, *F*_(1, 13)_ = 0.10, *p* > 0.05. There was no interaction between Group and training day, *F*_(1, 13)_ = 0.01, *p* > 0.05.

**Table 1:** **Mean and standard error of the mean (SEM) PreCS lever pressing across the first trial of Stage I, Stage II, and Test**.

**Group**		**Stage I**			**Stage II**		**Test**
		**1**	**2**	**3**	**1**	**2**	
Block-Sal	Mean	22.85	16.29	24.14	18.28	20.28	22.43
	SEM	3.67	2.10	4.21	4.99	3.33	5.69
Block-BUP	Mean	16.75	14.62	17.62	18.37	17.75	22.25
	SEM	4.33	2.28	4.19	2.19	2.82	1.92
Control-Sal	Mean				23.43	17.43	24.14
	SEM				6.39	5.77	4.42
Control-BUP	Mean				19.00	12.43	19.57
	SEM				1.40	3.59	2.11

Pre-CS lever pressing during the first trial across both training days of Stage II was not significant between groups, *Fs*_(1, 25) < 1_, *p* > 0.05. Furthermore, pre-CS lever pressing did not significantly change between the days of Stage II training when averaged across Groups, *F*_(1, 25)_ = 1.64, *p* > 0.05. There was no significant interactions between group, infusion and days of training, *Fs*_(1, 25) < 3_, *p* > 0.05.

There was no difference in pre-CS lever pressing across groups when averaged across trials of test, *Fs*_(1, 25)_ < 3, *p* > 0.05. There was however a significant linear decrease in pre-CS lever pressing across trials when averaged across Groups, *F*_(1, 25)_ = 10.60, *p* < 0.05, possibly due to satiety nearing the end of the session. Group by trial interactions were not significant, *Fs*_(1, 25)_ < 2, *p* > 0.05.

### Suppression ratios

The mean and *SEM* suppression ratios from Stage I, Stage II, and Test are shown Figure 1B. Inspection of the figure indicates comparable acquisition of conditioned fear across Groups Block-Bupivacaine and Block-Saline. This was supported by the analyses.

There was no overall difference in suppression ratios between Groups Block-Bupivacaine and Block-Saline, averaged across days, *F*_(1, 13)_ = 2.53, *p* > 0.05. There was a significant effect of days of Stage I training, averaged across groups, indicating the acquisition of conditioned fear to CSA, *F*_(1, 13)_ = 34.56, *p* < 0.05. There was no group × day interaction, *F*_(1, 13)_ = 0.12, *p* > 0.05.

During Stage II, groups Block continued to express fear to CSAB and groups Control acquired fear. There was a main effect of Stage I training, *F*_(1, 25)_ = 21.06, *p* < 0.05, so that groups Block expressed more fear than groups Control, during Stage II. There was no main effect of infusion (Bupivacaine vs. Saline), *F*_(1, 25)_ = 1.04, *p* > 0.05. This shows that reversible MIT inactivation had no significant effect on the expression of fear during Stage II. There was a main effect of day, *F*_(1, 25)_ = 22.38, *p* < 0.05, showing that fear increased from Day 1 to 2. This increase in fear was significantly greater for groups Control than groups Block because the group (Block vs. Control) × day interaction was significant, *F*_(1, 25)_ = 11.69, *p* < 0.05. This shows the acquisition of fear in groups Control. However, infusion of bupivacaine into the MIT had no significant effect on Stage II learning or fear expression, because the infusion (Bupivacaine vs. saline) × day interaction was not significant, *F*_(1, 25)_ = 0.71, *p* > 0.05. Finally, there was no 3 way interaction, *F*_(1, 25)_ = 2.08, *p* > 0.05.

Inspection of suppression ratios on test indicates the presence of blocking (i.e., high suppression ratio) in group Block-Saline and an attenuation of this blocking in group Block-Bupivacaine. Indeed, there was evidence for blocking of fear conditioning in saline treated animals because there was significantly less fear (i.e., suppression ratios were higher) in Group Block-Saline compared to the remaining three groups [*F*_(1, 25)_ = 5.7, p <0.05] or compared to group Control-Saline alone, *F*_(1, 25)_ = 7.11, *p* < 0.05. In contrast, there was no evidence for blocking in bupivacaine treated animals because there was no significant difference in fear between group Block-Bupivacaine vs. groups Control-Saline and Control-Bupivacaine, *F*_(1, 25)_ = 1.3, *p* > 0.05, or vs. Control-Bupivacaine alone, *F*_(1, 25)_ < 1, *p* > 0.05. Finally, there was no evidence that reversible inactivation of MIT had any effect on fear learning in the control group because group Control-Bupivacaine did not differ from group Control-Saline, *F*_(1, 25)_ < 1, *p* > 0.05.

## Discussion

Here we studied the role of MIT in the associative blocking of Pavlovian fear conditioning. In Stage I, rats in the blocking groups were trained to fear CSA. Then, in Stage II, CSA was presented in compound with a second CS, CSB, and paired with shock. The results showed that fear conditioning of CSA blocked fear learning to CSB. Less was learned about CSB in the block group than by a control group that did not receive Stage I training. Reversible inactivation of the MIT prior to Stage II training had no effect on the expression of fear during Stage II and had no effect on fear learning to CSB in the control group. Rather, reversible inactivation of MIT acted selectively to prevent associative blocking and enable normal fear learning to CSB.

These results show, for the first time that MIT is an important component of the neural circuitry for predictive fear learning. Reversible inactivation of MIT enabled rats in group Block-Bupivacaine to learn to fear the target CS which otherwise would not have been learned about. This strongly implicates MIT in the processes by which prediction error regulates fear learning. It is not immediately clear, though, how this may occur. Prediction error can act directly on association formation by determining the effectiveness of the shock reinforcer (Rescorla and Wagner, 1972). Unexpected or poorly predicted USs support more learning than expected or well-predicted USs. Imaging data from both rats and humans show that MIT activity is greater to unexpected than expected aversive USs (Dunsmoor et al., 2008; Furlong et al., 2010). This suggests that diminutions or reductions in MIT shock US processing are causal to blocking and, hence, that blocking should be prevented by manipulations that increase the activity of MIT neurons. Prediction error can also affect learning indirectly, by guiding attention to CSs that are better predictors of the US at the expense of CSs that are poorer predictors (Mackintosh, 1975; Pearce and Hall, 1980). The MIT may play an important role in allowing prediction error to influence the associability of CSs. This role would be more consistent with the historical suggestion that the MIT contribute to arousal and awareness (Groenewegen and Berendse, 1994).

There are at least three issues that bear on interpretation of these possibilities. First, the cannula placements in this experiment, while targeted at the CM, affected several distinct MIT regions including PVT, IMD, and Re. It remains to be determined whether there are differences between these regions, or between different cell types within these regions, in their contributions to predictive fear learning. Second, the MIT neurotransmitters and their receptors important for predictive fear learning are unknown. Here we used a sodium channel blocker to inactivate the MIT and this manipulation could affect not just CM and MIT neurons but also fibers of passage. The effects of more specific manipulations of MIT neurotransmitter function in predictive fear learning warrant further investigation. Finally, the reversible inactivation used here affected MIT during the CS presentations, US presentations, inter-trial intervals, and also the post-conditioning consolidation periods whereas previous imaging studies reflect activity over a shorter period of time. Which of these periods was critical for the observed effects here is unclear. Each of these caveats highlights the need for further research on the role of MIT in fear learning.

The absence of any effect of MIT inactivation in the Control-Bupivacaine group is also worthy of comment. It might be expected that if a manipulation prevents associative blocking then it should also augment the acquisition of fear in control animals. There was no evidence here for such an augmentation in group Control-Bupivacaine. It is possible that this absence of an effect in group Control-Bupivacaine was due to use of conditioned suppression as the measure of learned fear and that another measure of learned fear may have been more sensitive to such an augmentation. However, we consider this unlikely. Past research using other measures of learned fear, such as the species-typical defense response of freezing, also shows that manipulations which prevent blocking do not consistently affect conditioning to controls in the same experiment (McNally and Cole, 2006). We suggest that this finding reflects an advantage of the blocking design for studying prediction error. A key feature of the blocking design is that it allows systematic manipulation of prediction error to permit study of learning to a target CS under conditions where little or no learning would normally occur. This may render blocking more sensitive than simple acquisition of fear to manipulations affecting prediction error.

To the best of our knowledge, these are the first data implicating MIT in fear learning, and in predictive fear learning in particular. However, there have been previous assessments of the role of the posterior intralaminar thalamus (PIT) in fear conditioning, largely because of its potential as a site of convergence between auditory CS and aversive US information during conditioning (LeDoux et al., 1987; Campeau et al., 1997). Focal electrical stimulation of PIT and the immediately dorsolateral medial geniculate nucleus is able to serve as a US to condition autonomic responses to an auditory CS (Cruikshank et al., 2004). The effects of specific manipulations of the PIT on fear learning have been mixed. For example, Shi and Davis (1999) reported that PIT may be important in fear learning because it conveys US-related information. Specifically, Shi and Davis reported that combined electrolytic lesions of the PIT and insular cortex (Ins) impaired the acquisition of auditory fear conditioning and also disrupted behavioral reactivity to the footshock US. However, Brunzell and Kim (2001), using combined pre-training electrolytic lesions of PIT and Ins, reported no effect on the acquisition of auditory or contextual fear learning. Finally, Lanuza et al. (2008) reported that whereas electrolytic lesions of PIT disrupted fear conditioning when a shock US was used, such lesions did not disrupt this learning when a loud noise US was used, and importantly, excitotoxic lesions of the same PIT region had no effect on fear learning with a shock US (see also Campeau et al., 1997). These findings suggest that although shock US-related information may pass through the PIT, fear conditioning does not necessarily involve PIT itself. It is worth emphasizing that the present experiment differed from this past research in that the MIT regions studied here were considerably anterior (at least 3 mm) and medial to the PIT regions studied previously. The connectivity of these two intralaminar regions differ with, among other differences, the PIT providing stronger direct projections to the amygdala than the MIT (LeDoux et al., 1990; Romanski and LeDoux, 1992).

Recent electrophysiological behavioral evidence implicates MIT in expression of learned fear. Firstly, somatostatin-positive CeAl neurons project directly to MIT and excitatory synaptic transmission in these neurons is potentiated by fear conditioning (Penzo et al., 2014). Secondly, reversible inactivation of MIT via infusions of the GABA agonist muscimol impairs the expression of fear, as measured by freezing, when rats are tested 24 h after conditioning but not when they are tested at shorter intervals (Padilla-Coreano et al., 2011). Finally, recent findings show that the posterior PVT (pPVT), in particular, is important for expression of learned fear as measured by freezing or conditioned suppression (Li et al., 2014). During Stage II of this experiment, animals in groups Block expressed high, asymptotic levels of conditioned fear. Reversible inactivation of MIT had no effect on this expression of learned fear. The reasons for these differences in the effects of MIT inactivation on fear expression in different experiments are unclear. One possibility is that MIT contributes to expression of some but not all measures of learned fear. Here we used conditioned suppression as a measure of learned fear whereas Padilla-Coreano et al. (2011) measured the species-typical defense response of freezing. Similar differences in impact on expression of freezing vs. conditioned suppression have been reported for manipulations of other brain regions (Amorapanth et al., 1999). We consider this unlikely because Li et al. (2014) have shown that PVT is important for expression of fear as measured via freezing or conditioned suppression. More likely is the possibility is that distinct MIT regions serve distinct roles in fear learning and expression. The pPVT is selectively important for expression of learned fear (Li et al., 2014). The pPVT is well-placed to influence fear expression because of its dense projections to the CeA. Here we studied more anterior sections of MIT, focusing on CM, and show an important role for CM in predictive fear learning. The CM, in contrast to PVT, has sparse connections with CeA and may not influence fear expression (Su and Bentivoglio, 1990) but is important for learning. It will be important to continue to identify the specific functions of the MIT in Pavlovian fear learning.

### Conflict of interest statement

The authors declare that the research was conducted in the absence of any commercial or financial relationships that could be construed as a potential conflict of interest.
